# Protocol for a multicentre randomiSed controlled TRial of IntraVEnous immunoglobulin versus standard therapy for the treatment of transverse myelitis in adults and children (STRIVE)

**DOI:** 10.1136/bmjopen-2015-008312

**Published:** 2015-05-25

**Authors:** M Absoud, J Gadian, J Hellier, P A Brex, O Ciccarelli, G Giovannoni, J Kelly, P McCrone, C Murphy, J Palace, A Pickles, M Pike, N Robertson, A Jacob, M Lim

**Affiliations:** 1 Department of Children's Neurosciences, Evelina Children's Hospital at Guy's and St Thomas’ NHS Foundation Trust, King's Health Partners Academic Health Science Centre, London, UK; 2 Department of Biostatistics, Institute of Psychiatry, Psychology and Neuroscience, King's College London, London, UK; 3 Department of Neurology, King's College Hospital NHS Foundation Trust, King's Health Partners Academic Health Science Centre, London, UK; 4 UCL Institute of Neurology, Queen Square, London, UK; 5 Centre for Neuroscience and Trauma, Blizard Institute, University of London and Bart's Health NHS Trust, London, UK; 6 King's Clinical Trials Unit, Institute of Psychiatry, Psychology and Neuroscience, King's College London, London, UK; 7 Centre for the Economics of Mental and Physical Health, Institute of Psychiatry, Psychology and Neuroscience, King's College London, London, UK; 8 Department of Neurology, Oxford University Hospitals NHS Trust, Oxford, UK; 9 Department of Paediatric Neurology, Oxford University Hospitals NHS Trust, Oxford, UK; 10 Institute of Psychological Medicine and Clinical Neurosciences, Cardiff University and Cardiff and Vale NHS Trust, Cardiff, UK; 11 The Walton Centre, Walton Centre NHS Foundation Trust, Liverpool, UK

**Keywords:** HEALTH ECONOMICS, IMMUNOLOGY, NEUROLOGY, STATISTICS & RESEARCH METHODS

## Abstract

**Introduction:**

Transverse myelitis (TM) is an immune-mediated disorder of the spinal cord which causes motor and sensory disturbance and limited recovery in 50% of patients. Standard treatment is steroids, and patients with more severe disease appear to respond to plasma exchange (PLEX). Intravenous immunoglobulin (IVIG) has also been used as an adjunct to steroids, but evidence is lacking. We propose the first randomised control trial in adults and children, to determine the benefit of additional treatment with IVIG.

**Methods and analysis:**

170 adults and children aged over 1 year with acute first episode TM or neuromyelitis optica (with myelitis) will be recruited over a 2.5-year period and followed up for 12 months. Participants randomised to the control arm will receive standard therapy of intravenous methylprednisolone (IVMP). The intervention arm will receive the above standard therapy, plus additional IVIG.

Primary outcome will be a 2-point improvement on the American Spinal Injury Association (ASIA) Impairment scale at 6 months postrandomisation by blinded assessors. Additional secondary and tertiary outcome measures will be collected: ASIA motor and sensory scales, Kurtzke expanded disability status scale, International Spinal Cord Injury (SCI) Bladder/Bowel Data Set, Client Services Receipt Index, Pediatric Quality of Life Inventory, EQ-5D, SCI Pain and SCI Quality of Life Data Sets. Biological samples will be biobanked for future studies. After 6-months' follow-up of the first 52 recruited patients futility analysis will be carried out. Health economics analysis will be performed to calculate cost-effectiveness. After 6 months’ recruitment futility analysis will be performed.

**Ethics and dissemination:**

Research Ethics Committee Approval was obtained: 14/SC/1329. Current protocol: v3.0 (15/01/2015). Study findings will be published in peer-reviewed journals.

**Trial registration numbers:**

This study is registered with EudraCT (REF: 2014-002335-34), Clinicaltrials.gov (REF: NCT02398994) and ISRCTN (REF: 12127581).

**Strengths and limitations of this study**

- The first randomised multicentre UK trial in children and adults with transverse myelitis or neuromyelitis optica, recruiting 170 patients over 2.5 years with a 12-month follow-up period.
- Outcome measures will include motor, sensory, functional and quality of life measurements by blinded assessors.
- Health economics analysis will include health and social care costs.
- Findings may inform treatment decisions in other rare, inflammatory central nervous system disorders.
- High recruitment rate required due to low incidence of condition.

## Introduction

Transverse myelitis (TM) is a rare inflammatory disorder of the spinal cord affecting approximately 350 children and adults annually in the UK.^1^ ^2^ Histologically, TM is characterised by spinal cord immune cellular infiltration, and pathogenesis is mediated by a variety of immunological mechanisms.^3^ Clinical features include a rapid onset of motor, sensory and autonomic dysfunction, and a prolonged recovery phase which may continue for up to 4 years.^4^ Diagnostic criteria for TM were established by the TM Consortium Working Group in 2002, to distinguish TM from other conditions including MS and a clinically isolated syndrome.^5^ A proportion of patients initially diagnosed with TM will subsequently relapse, often with involvement of other parts of the central nervous system, and may often be diagnosed with either multiple sclerosis (MS) or neuromyelitis optica (NMO). However, a proportion of patients remain as relapsing TM of as yet unknown aetiologies.

NMO is a relapsing subset of TM, caused by antibodies to aquaporin-4, an astrocytic water channel.^6^ Clinically, patients have predominantly recurrent episodes of myelitis and optic neuritis. Initial presentation may be with myelitis alone, making it clinically and radiologically indistinguishable from TM, and patients are thus subjected to the same acute therapeutic strategies.

The American Spinal Injury Association (ASIA) international standards for neurological classification of spinal cord injury enables a standardised assessment of neurological outcome, where A is no sensory or motor function in S4–5, and E is normal function.^7^ Retrospective data from paediatric cohorts suggest that 30% have ASIA A-C or expanded disability status scale (EDSS) ≥4 after TM,^8^ and a retrospective French multicentre study in adults found that 36% had a poor prognosis as defined by death or non-ambulatory status with current therapy.^9^ Since this is primarily a disease of younger people, this results in significant cumulative demands on health and social care resources.

There are no robust controlled trials in children or adults to inform on the optimal treatment of TM. Standard treatment with intravenous methylprednisolone (IVMP) is based on class IV evidence that it shortens relapse duration and speeds recovery in exacerbations of adult multiple sclerosis.^6^ ^10–12^ Given the disease severity and poor outcomes, plasma exchange (PLEX) has been used in addition to standard therapy. Addition of PLEX showed benefit in two studies: a retrospective analysis of 122 adults with TM;^10^ and a small randomised controlled trial (RCT) in adults with a steroid unresponsive acute central nervous system (CNS) demyelination which included four patients with TM.^13^ However, PLEX is not universally available in the NHS, particularly at short notice and on weekends, and can be technically difficult and costly to administer.^14^

Randomised controlled trials have demonstrated IVIG efficacy in a number of neurological conditions.^15^ In steroid-unresponsive CNS demyelination, IVIG is often used, although supporting data are limited to small case series and single case reports.^16^ ^17^ IVIG appears to inhibit complement binding, neutralise pathogenic cytokines, downregulate antibody production, enhance remyelination and modulate phagocytosis and T-cell function.^18^ The majority of these factors are common across inflammatory disorders of the CNS including TM,^19^ providing a strong rationale for its use. The availability, ease of administration, familiarity and safety also make IVIG an attractive option in the acute setting.

### Trial objectives and design

This multicentre, single blind, parallel group RCT will generate evidence to inform clinical and health economic decisions regarding IVIG use in adults and children with TM.

### Primary objective

1. To evaluate if additional and early treatment with IVIG is of extra benefit in TM when compared to the current standard therapy of IVMP.

### Secondary objectives

1. The clinical and paraclinical data collected from patients will provide a robust resource and platform for other clinical studies, including identification of early predictors of poor outcome.
2. Biobanked samples from patients recruited to the study will be collected and used for carefully designed biological studies by a consortium of established basic science researchers in the field.

## Methods

### Study setting

Treatment and follow-up will be in the participating tertiary neurology centres, and recruitment will also occur from feeder district general hospitals and rapid general practitioner (GP) referrals. For further details on participating centres and trial registration, please see online supplementary appendix 1.

### Eligibility criteria

#### Inclusion criteria

Patients must satisfy all inclusion criteria to be eligible for recruitment. They will be eligible if all of the following apply at the time of randomisation:

1. Age 1 year or over
2. Diagnosis of EITHER acute first onset transverse myelitis (using the TM Consortium Working Group 2002 criteria^6^)—patients must fulfil all of the following criteria:
  - Sensory, motor or autonomic dysfunction attributable to spinal cord disease
  - Bilateral signs and/or symptoms (not necessarily symmetric)
  - Sensory level (except in young children <5 years where this is difficult to evaluate)
  - Lack of MRI brain criteria consistent with multiple sclerosis^20^
  - Progression to nadir between 4 h and 21 days
  OR first presentation of neuromyelitis optica (using standardised criteria^21^)—patients must fulfil both absolute criteria:
  - Optic neuritis
  - Acute myelitis
  plus two out of three supportive criteria (as AQP4 is often not available acutely, only the first two supportive criteria would be applied):
  - Brain MRI not meeting criteria for MS at disease onset
  - Spinal cord MRI with contiguous T2-weighted signal abnormality extending over three or more vertebral segments, indicating a relatively large lesion in the spinal cord
  - Aquaporin 4 IgG seropositive status
3. ASIA Impairment Score of A-C
4. Randomisation to occur no later than day 5 of steroids, and, if definitely known, within 21 days from symptom onset.
5. Give assent (8–16 years)/consent to participate in the trial.

#### Exclusion criteria

In addition to failing to meet the inclusion criteria, exclusion criteria include any of the following:

1. Contraindication to IVIG as stated in the product SmPC, or receiving IVIG for other reasons
2. Previously known systemic autoimmune disease (eg, systemic lupus erythematosus) or any evidence of systemic inflammation during current presentation.
3. Direct infectious aetiology (eg, varicella zoster)
4. Previous episode of CNS inflammatory demyelination
5. Acute disseminated encephalomyelitis (ADEM)
6. Other causes of myelopathy not thought to be due to myelitis (eg, nutritional, ischaemic, tumour, etc)
7. Other disease which would interfere with assessment of outcome measures
8. Known pregnancy
9. Circumstances which would prevent follow-up for 12 months.

### Interventions

Patients randomised to the **control arm** of this study will be prescribed IVMP. Paediatric patients will receive 30 mg/kg or 500 mg/m^2^ capped to a maximum dose of 1 g/day for 5 days. Adult patients will be given 1 g/day for 5 days. Clinicians may follow this with an oral steroid taper according to local practice.

Patients randomised to the **intervention arm** will receive the above standard therapy *plus* additional IVIG at a total dose of 2 g/kg. Doses will be divided over 2 days (children <41.2 kg) or 5 days (all other patients) and individual doses may vary slightly to minimise drug wastage and anticipate for difficult intravenous access in small children.

Treatment failure will be defined as no improvement 14 days after presentation and/or 5 days after completion of treatment, and will be documented. Rescue therapy may be initiated at this point. Given the therapeutic effect of PLEX, treatment will be standardised to comprise five cycles in which at least 75% of plasma volume is exchanged, with a gap of 24–48 h between cycles. An additional course of IVMP may be given if there is a delay between the decision to start PLEX and therapy initiation, at the discretion of the treating clinician. The duration and intensity of neurorehabilitation input will be recorded to enable comparison between groups.

### Outcome measures

Outcome measures have been selected to give a ‘hard’ clinical end point that will have clinical significance, and will be assessed at the local centre by a blinded assessor. To minimise loss to follow-up, assessments are timed to coincide with routine clinical follow-up. All outcome measures are internationally accepted scales, and the primary outcome measure is the ASIA Impairment scale, which is used to measure disability in TM.^22^ A 6-month time point has been selected, as the majority of neurological recovery is likely to have occurred by this point. Additional data points will be taken at 3 and 12 months to aid statistical analysis.

#### Primary outcome measure

- A two point or greater improvement in the ASIA scale (classified A-E) at 6 months postrandomisation, when compared to baseline, will indicate a positive outcome.

#### Secondary outcome measures

- A change in the ASIA motor scale (0–100) and sensory scale (0–112)
- A change in the Kurtzke expanded disability status scale (EDSS) with Neurostatus scoring
- EQ-5D-Y (patients aged 8–12 years at presentation) or EQ-5D-5 L (patients aged ≥13 years at presentation)
- International SCI Quality of Life Basic Data Set (patients aged ≥13 years)
- Client Service Receipt Inventory (CSRI).

#### Tertiary outcome measures

- International SCI Bladder/Bowel Data Set (patients aged ≥13 years)
- International SCI Pain Basic Data Set (patients aged ≥13 years)
- Pediatric Quality of Life Inventory TM (PedsQL Parent Report for Toddlers; patients aged 2–4 years)
- Pediatric Quality of Life Inventory TM (PedsQL Parent Report for Young Children; patients aged 5–7 years).

### Participant timeline

Patients will be enrolled to the study for 1 year (table 1).

**Table 1:** Timeline of trial interventions

Schedule Treatment day (TD) Time point (T)	T0 (Screening, baseline and prediagnosis tests)	T1 (Treatment and discharge)							T2 3M	T3 6M	T4 12M	Withdrawal
		TD 1*	TD 2	TD 3	TD 4	TD 5	Rescue therapy†	Discharge				
Screening with diagnostic algorithm and core investigations including physical examination	X											
Patient information and informed consent	X											
Eligibility form	X											
Registration form	X											
Prediagnosis tests—for example, MRI and AQP4	X											
Randomisation	X											
Biobank samples	X									X		
ASIA Impairment Score (A-E)	X						X	X	X	P	X	
ASIA Motor and Sensory Score	X						X	X	X	S	X	
Neurostatus scoring (Kurtzke functional systems and EDSS)	X							X	X	S	X	
8–12 years EQ-5D-Y	X								X	S	X	
≥13 years EQ-5D-5L	X								X	S	X	
≥13 years SCI QoL Basic data set									X	S	X	
CSRI									X	S	X	
≥13 years SCI Bladder										T	X	
≥13 years SCI Bowel										T	X	
5–7 years Peds QL										T	X	
2–4 years Peds QL										T	X	
Treatment form						X						
Concomitant medications								X	X	X	X	
Discharge form								X				
Rescue therapy form (if needed)†							X					
Relapse form (at any time point if needed)†								X	X	X	X	
Adverse events‡		X	X	X	X	X						
Study Status Form									X	X	X	
Withdrawal form (at any time point)†												X

*Treatment day 1: IVIG treatment (if applicable) to start on the same day as randomisation. Steroids may be started up to 5 days prior to randomisation.

†Rescue therapy, relapse and withdrawal forms may only be necessary for a small subset of patients.

‡Adverse events will be collected throughout the study.

ASIA, American Spinal Injury Association; AQP4, aquaporin-4; CSRI, Client Service Receipt Inventory; EDSS, expanded disability status scale; Peds QL, Pediatric Quality of Life Inventory; P, primary outcome measure; S, secondary outcome measure; SCI, Spinal Cord Injury; T, tertiary outcome measure.

### Trial duration

The project will take 3.5 years. Patient recruitment will take place over the first 30 months, and collection of data will continue until 42 months.

### Sample size

The power analysis has taken into account the inclusion of a futility analysis to be undertaken after recruitment of one-third of the target sample. We have assumed that the proportion of participants showing a two-point improvement (or greater) on the ASIA Impairment scale will be approximately 0.5 (50%) in the control arm and a minimum of 0.75 (75%) in the intervention arm, based on available data from epidemiological studies.^2^ ^7^ The sample size calculation is based on the conservative assumption of no correlation between repeated measures. Under these assumptions, 76 patients per group will provide 90% power and 5% type II errors for a two-sided test. The sample size will be inflated for attrition; on the basis of our experience and the design which minimises loss to follow-up, we estimate 10% attrition. This requires a recruitment of 170 patients (85 participants per arm).

The ASIA total motor score (0–100) is a secondary outcome. Stata ‘*sampsi*’ indicates that using an Analysis of Covariance with a baseline to end point correlation of 0.6, there will be 87% power to detect a difference between the control and treatment arms of a medium to large effect size of 0.4. Such a difference will be of clinical significance.

### Recruitment plan

TM has an estimated UK incidence of 350/year, and the study centres cover approximately half of the UK population. Assuming a recruitment rate of 39% over a period of 2.5 years, we would expect to recruit 170 patients. This recruitment rate is based on a patient and public consultation within the TM society, given that patients will be admitted with a devastating weakness, and the control arm will be receiving standard intervention.

### Randomisation

Prior to randomisation but after consent, site staff will register a recruit on the web-based electronic data capture system (InferMed MACRO), hosted at the King's Clinical Trials Unit (KCTU). Each will be assigned a unique study patient identification number (PIN) by the system. Once baseline assessments are complete, the trained trial staff will access the KCTU randomisation system and randomise the patient at the individual level using stratified block randomisation by service type (adult or child); the block will randomly vary in size. Treatment allocation will be at a ratio of 1:1. Randomisation will occur during office hours when IVIG is available. Patients eligible for trial recruitment presenting outside of these times will be started immediately on IVMP and then randomised at the first available opportunity.

### Blinding

Owing to the technical challenges of masking IVIG from saline, the need for rapid recruitment and the fact that follow-up will be many months after the event using objective, well-defined clinical end points, treatment will not be blinded. Staff carrying out study assessments and statistical analyses will be blinded to intervention.

### Data collection methods

**Figure 1:**
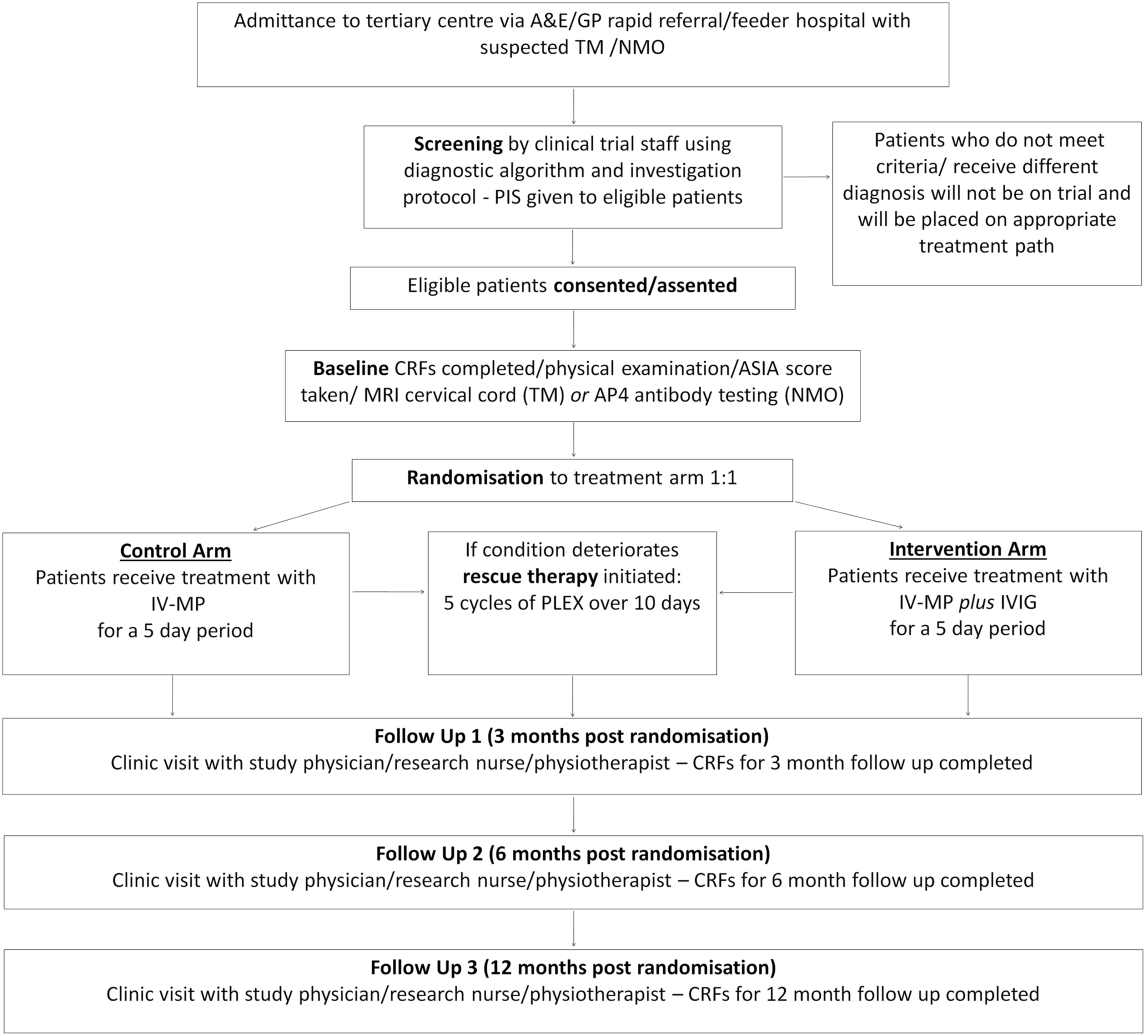
Flow chart showing the process of patient recruitment, treatment and follow-up.

The Kurtzke neurological examination, ASIA Impairment Score, ASIA Motor and Sensory Score and EDSS scores will be completed, together with the treatment, discharge and follow-up forms (see figure 1). All assessments should ideally be performed by the same blinded assessor, who may be a study physician, physiotherapist or research nurse. All assessors will have successfully completed ASIA and EDSS online training modules (further information is detailed in online supplementary appendix 1). Biological samples will be collected, processed and stored as detailed in online supplementary appendix 3.

### Withdrawal

Patients may withdraw at any time. All withdrawals from randomised treatment will be reported. If consent is given, any existing data or samples will be retained, and follow-up data will continue to be collected; if not, then a withdrawal form will be completed. The investigator may withdraw patients from the study drug in the event of intercurrent illness, AEs, SAEs and SUSARs, subsequent evidence of a different aetiology, protocol violations, cure, administrative or other reasons. All data will be analysed on an intention-to-treat basis.

### Study data

#### Data management

Data will be managed using the InferMed MACRO database system. An electronic case report form (eCRF) will be created using the InferMed Macro system. This system is regulatory compliant (GCP, 21CRF11, EC Clinical Trial Directive). The eCRF will be created in collaboration with the trial statisticians and the chief investigator (CI) and maintained by the King's Clinical Trials Unit. It will be hosted on a dedicated secure server within KCL. Source data will be entered by authorised staff onto the eCRF with a full audit trail.

#### Database passwords

Database access will be strictly restricted through passwords to the authorised research team. The CI or delegate will request usernames and passwords from the KCTU. It is a legal requirement that passwords to the eCRF are not shared, and that only those authorised to access the system are allowed to do so. If new staff members join the study, a personalised username and password will be requested via the CI or delegate.

#### Identifiable data

All participant contact information data will be stored on spreadsheets within the recruiting NHS site, which will have restricted access from password protected computers. Accrual data uploaded to the UKCRN portfolio database will be anonymised and collated by the CI or delegate to the CLRN. No identifiable data will be entered on the eCRF or transferred to the KCTU. Participants will be identified on the study database using a unique code and initials. The investigator will maintain accurate patient records detailing observations on each patient enrolled.

### Statistical methods

#### Primary analysis

If the study continues to full recruitment, analyses of effectiveness will be pragmatic, based on the intention-to-treat (ITT) sample. The significance level will be 5% (two-sided) for specified analyses. The estimated effect size and its precision (95% CIs) will be presented for all outcomes. The main statistical analyses will establish the effectiveness of IVIG against standard therapy at 6 months postrandomisation (see primary end points above). To this end, linear mixed modelling (LMM) will be employed. In such models, the binary outcome variable measured at the post-treatment time points (3, 6 or 12 months) features as the dependent variable with outcome at baseline (if applicable), stratification factors (service level), treatment arm and a treatment x time interaction term included as covariates. To account for correlation between repeated measures on the same individual, a subject-varying random intercept will be included. Mixed effects logistic regression can be completed using the ‘*xtmelogit’* command in Stata.

There are expected to be some missing data in the post-treatment outcome variables. The LMM analyses are based on maximum likelihood and will provide valid inferences under a missing at random (MAR) missingness mechanism.

#### Secondary analyses

The secondary clinical assessments (EDSS, ASIA motor and sensory scales, SCI data sets, PedsQL, EQ5D and CSRI) with repeated measurements will also be analysed within a LMM framework where generalisations of the LMM will be utilised to allow for outcomes with non-normal data if necessary. Those measures with one follow-up assessment will be evaluated with in a Generalised Linear Model. The dependent variable will feature as the outcome measure. The covariates will be the corresponding baseline measure (if applicable), stratification factors and treatment group.

#### Exploratory moderator analyses

All analysis will be repeated considering age status (adult or child) as a moderator in interaction with the treatment group (control or intervention), allowing estimates of treatment effect in the subpopulations to be summarised.^23^ We will carry out further explanatory analyses to assess the efficacy of the treatment within NMO or idiopathic TM diagnosis and further putative biological markers by allowing for interactions with the treatment arm. When considering these moderator analyses, following established methods^24^ we will centre and orthogonalise interaction terms.

Further information on the statistical analysis plan can be found in the protocol and online supplementary appendix 1.

#### Interim futility analysis

TM is a rare disease and therefore requires a multicentre trial spanning several years, precluding recruitment to other interventional studies for this cohort. As such, an interim futility analysis will be performed after a 6-month follow-up of 52 patients. If sample sizes are equal, the trial may be abandoned if the successes in the intervention group are fewer than in the standard group. The probability of abandoning the study at the interim analysis is 0.4449 if there is no difference between treatment groups, and 0.0201 if treatment improves outcome. The primary trial statistician will remain blinded to the intervention and control groups, and therefore an additional unmasked trial statistician will perform the interim analysis. The results will be reviewed by the Data Monitoring Committee, which has the ability to terminate the trial prematurely.

### Economic analysis

Drug pricing data and primary care, secondary care and social care costs will be calculated as previously described. Costs will be combined with the primary outcome measure in the form of a cost-effectiveness analysis. If IVIG results in higher costs and better outcome, then an incremental cost-effectiveness ratio will be generated to show the extra cost incurred to achieve an extra unit of improvement. Owing to the uncertainty around results, cost-effectiveness planes and cost-effectiveness acceptability curves will be used, with bootstrapping of skewed results.

Long-term cost-effectiveness over 5-year and 10-year periods will be calculated using a Markov model. Response to treatment will be classified, and transition probabilities between groups will be derived from 6-month and 12-month follow-up data. Costs and QALYs for each category will be derived from the trial data. As limited data will be available on long-term costs, we will conduct both deterministic and probabilistic sensitivity analyses.

All causes of withdrawal from randomised treatment will be reported. χ^2^ (Fisher's exact test) will be used for categorical outcomes (eg, serious adverse events and mortality).

There will be missing data in post-treatment outcome variables as participants discontinue treatment or are lost to follow-up. Inferences will be valid provided the missing data generating mechanism is missing at random (MAR), and is not predicted by any variables in the model, that is, missingness is predicted only by variables that are included in the model.

### Economic evaluation

The use of IVIG, IVMP, additional treatments and rescue PLEX will be recorded throughout the follow-up period and costed using drug pricing data from the British National Formulary and the Department of Health. Use of primary care, secondary care and social care will be recorded at three-month, 6-month and 12-month follow-ups using the CSRI, and costs calculated to determine total cost for THE control and treatment arms.

A cost-effectiveness analysis will be performed using the primary and secondary outcome measures of improvement in ASIA scores, and secondary outcome of QALYs with EQ-5D-Y, EQ-5D-5L and CSRI. If IVIG results in higher costs and better outcome, then an incremental cost-effectiveness ratio will be generated to show the extra cost incurred to achieve an extra unit of improvement.

### Data monitoring

The Data Monitoring Committee (DMC) will review effectiveness and safety data during the trial to inform their recommendations to the Trial Steering Committee (TSC). The DMC is independent from the sponsor and funders, and will consist of a statistician, a clinician and a clinician scientist.

### Harms

Most adverse drug reactions that occur in this study, whether serious or not, will be expected treatment-related side effects as IVIG has a well-established side effect profile. Monitoring and reporting of adverse events will be performed by the site PI and research team, and will be recorded on an eCRF and uploaded to the InferMed MACRO database. Serious adverse events will be reported expeditiously to the sponsor, who in turn will inform the CI. The CI will assess SUSAR status. If a SUSAR is detected, the CI will work with the sponsor to report to the regulatory authorities. The CI will report to the relevant ethics committees and DMC.

An independent TSC and DMC will convene every 6 months; the TSC's key purpose will be to monitor study progress and act on the recommendations of the DMC. The DMC will aim to meet 3 weeks prior to the TSC convening. Additional meetings will be arranged if recommended by the DMC or TSC.

### Auditing

Monitoring of this trial will ensure compliance with Good Clinical Practice (GCP). Scientific integrity will be managed and oversight retained by the King's Health Partners Clinical Trials Office Quality Team. The investigator sites will provide direct access to all trial-related source data/documents and reports for the purpose of monitoring and auditing by the sponsor and inspection by local and regulatory authorities. Data will be evaluated for compliance with the protocol and accuracy in relation to source documents.

Following written standard operating procedures, the monitors will verify that the clinical trial is conducted and data are generated, documented and reported in compliance with the protocol, GCP and the applicable regulatory requirements.

## Ethics and dissemination

### Ethical and safety considerations

This trial has been approved by the UK National Research Ethics Service (NRES) committee (South Central—Berkshire B; REC 14/SC/1329). Clinical trial authorisation for a type A trial has been granted via the Medicines and Healthcare Products Regulatory Agency (MHRA) notification scheme. Written approval from the respective Research and Development (R&D) departments will be obtained for each participating site.

The CI will ensure that this study (and all subsequent approved amendments) is conducted in accordance with the principles of the Declaration of Helsinki (1996), in full conformity with the International Conference on Harmonisation of Technical Requirements for Registration of Pharmaceuticals for Human Use (ICH) Guidelines for GCP (CPMP/ICH/135/95 July 1996), the Research Governance Framework, and the Medicines for Human Use (Clinical Trial) Regulations 2004. Pharmacovigilance will be monitored by the CI, who will report to the REC, MHRA and funders (NIHR) during and at the end of the trial.

All protocol modifications will be disseminated to all relevant parties.

### Informed consent

A member of the trial staff will screen the patient and, if eligible, patients will be given age appropriate patient information sheets (PIS) explaining the trial (see online supplementary appendix 2). Trial staff will obtain informed written consent from patients over 16 years of age wishing to participate. For those aged under 16, assent will be obtained from the patient and consent from the parents or legal guardian. At the same time, consent will be obtained for the storage and use of participant data and biological specimens in future studies.

### Confidentiality

Only anonymised data will be entered onto the eCRF. Source data will be retained on password-protected Trust computers and in patient notes to protect patient confidentiality.

### Access to data

The CI will control access to data.

### Dissemination plan

Study findings will be presented in conferences and published in peer-reviewed journals.
